# Effectiveness of a group intervention to enhance resilience and improve the well-being of family caregivers of dementia patients

**DOI:** 10.3389/fpsyt.2025.1702324

**Published:** 2025-11-26

**Authors:** Sara Loma-Ramos, Elena Fernández-Martínez, Alberto González-García, Laura Bello-Corral, Leticia Sánchez-Valdeón

**Affiliations:** 1 Primary Care Management (SACYL), Armunia Health Centre, León, Spain; 2 Faculty of Health Sciences, Nursing and Physiotherapy Department, HeQoL Research Group, University of León, León, Spain; 3 Faculty of Health Sciences, Nursing and Physiotherapy Department, University of León, León, Spain

**Keywords:** resilience, dementia, caregiver, health education, public health, nurses, community health

## Abstract

**Introduction:**

Resilience is a key psychological resource for managing the emotional and physical demands associated with caring for people with dementia. It promotes psychological stability and protects caregiver health. However, evidence on structured interventions specifically designed to strengthen resilience in this population remains scarce. This study aimed to evaluate the effectiveness of a group-based intervention to enhance resilience in dementia caregivers, primary outcome, and to determine whether effects persisted three months post-intervention. Caregiver burden, coping strategies, and social support were examined as secondary outcomes.

**Methods:**

A quasi-experimental study with a single-group pre-test/post-test design was conducted with data collected at three time points: baseline (T1), post-intervention (T2), and three months post-intervention (T3). Sociodemographic characteristics were recorded, and resilience was measured using the CD-RISC 10. Caregiving burden was assessed with the Zarit Burden Interview, coping strategies with the CSQ Questionnaire, and perceived social support with the MOS Social Support Survey. The Wilcoxon signed-rank test was used for multiple comparisons (T1–T2–T3).

**Results:**

A significant increase in resilience was observed at post-intervention (T1–T2; p = 0.036), maintained at three-month follow-up (T1–T3; p = 0.040). Significant differences were found in caregiver burden (Zarit) between T2–T3 (p = 0.033), and in coping strategies: problem-solving (T2–T3, p = 0.032; T1–T3, p = 0.021), open emotional expression (T1–T2, p = 0.011; T1–T3, p = 0.041), and use of religion (T1–T2, p = 0.026). No significant changes were found in perceived social support.

**Conclusions:**

The intervention effectively enhanced resilience, improved adaptive coping strategies, and reduced caregiver burden. Healthcare professionals are encouraged to promote such interventions to support the well-being of dementia caregivers.

## 1 Introduction

The global increase in life expectancy has led to a higher prevalence of chronic diseases, among which dementia is particularly prominent (1). Dementia is understood as a severe neurodegenerative disorder due to its impact on cognitive functions such as thinking, speech, language, orientation, comprehension, judgement, planning, calculation, and learning ability, as well as a progressive loss of functional abilities. As a result, individuals affected require assistance from others to continue with daily life (2, 3). According to the World Health Organization (WHO), there are currently 57 million people diagnosed with dementia, with Alzheimer’s disease being the most common form, accounting for 60–70% of all cases (3).

Providing care for vulnerable individuals is an intrinsic human activity (4). From the moment a person is diagnosed with dementia, the family typically becomes the primary source of care. Within the family, the role of the main caregiver is usually established based on the relationship to the individual, perceived responsibility, and in some cases, legal obligation (5). This role is predominantly assumed by women, particularly spouses and daughters (6–12).

The role of caregiving often arises unexpectedly and with limited prior knowledge. Consequently, care tends to be provided through trial and error, based on personal experience, while caregivers assume responsibilities that demand continuous emotional and time investment. However, they frequently neglect their own well-being and often seek help only when confronted with extreme or emotionally unsustainable situations (5). Current theoretical frameworks (13, 14) highlight the importance of facilitating factors throughout the caregiving process, among which resilience stands out. Resilience is a multidimensional construct influenced by emotional, psychological, social, and environmental factors (15). Promoting resilience has been shown to reduce perceived burden, stress levels, and depressive symptoms, while enhancing quality of life and psychological well-being. According to Cerquera et al. (7), resilience can be defined as the capacity for personal growth developed when an individual is compelled to face an adverse situation, not only gaining benefits from the experience but also avoiding negative health consequences.

Resilience emerges as a key psychological resource that enables caregivers to adapt to changing demands, mitigate stress, and promote personal growth. Resilience is a multidimensional construct shaped by emotional, psychological, social, and environmental factors, and its promotion has been associated with lower burden, reduced depressive symptoms, and improved quality of life (16).

In line with Lazarus and Folkman (17) Transactional Model of Stress, the study by Duodu et al. (18), which focused on factors associated with resilience in caregivers of individuals with Alzheimer’s disease, identified a relationship between the development of coping strategies and higher levels of resilience. These strategies equip caregivers with the resources needed to face the challenges and pressures associated with their role.

Psychoeducational programmes (19–24) have consistently demonstrated benefits for caregivers’ quality of life. However, despite the benefits of psychoeducational interventions for caregivers, relatively few studies have specifically targeted resilience as their primary outcome (8, 25, 26). Given these gaps, there is a need for structured programmes designed to strengthen resilience in dementia caregivers. Therefore, the aim of this study is to evaluate the effectiveness of a group-based health promotion programme to foster resilience in caregivers and to examine the persistence of its effects three months after the intervention.

## 2 Methods

### 2.1 Study design

A quasi-experimental design was employed in this study, with participants selected through purposive convenience sampling. A structured psychoeducational intervention was implemented over six sessions, with data collected at three time points: prior to the first session (T1), immediately after the final session (T2), and three months post-intervention for follow-up (T3).

Given the primary aim of assessing the impact of the intervention, a single-group pretest-posttest design was selected to allow for the observation of changes within the group before and after the intervention.

The use of a single-group pretest–posttest design was justified by ethical and logistical considerations: random assignment to a control group without support could have increased caregiver burden and limited participation feasibility. This approach enabled the observation of changes over time within the same participants under naturalistic community conditions. Nevertheless, the absence of a control group restricts causal inference, as improvements might also reflect temporal adaptation, repeated testing, or social desirability bias.

### 2.2 Participants

The study population consisted of 29 primary female caregivers affiliated with the Alzheimer’s Association between 2023 and 2024.

Inclusion criteria were: woman, being over 18 years of age, providing unpaid care to a person with dementia, signing the informed consent form, and being available to participate in all three stages of the intervention.

The decision to exclusively include women was based on the higher prevalence of caregiving among females in this context, thereby justifying the specific focus on this group. Moreover, including only women reduced variability associated with biological and psychosocial sex-related factors, allowing for more controlled and homogeneous analysis and increasing the internal validity of the study.

### 2.3 Variables and instruments

The perception of caregiver well-being was assessed through a range of variables. Sociodemographic characteristics were included as independent variables. Dependent variables comprised resilience (primary outcomes), perceived caregiver burden, coping strategies for stress, and perceived social support (secondary outcomes).

Data were collected using an assessment battery consisting of an *ad hoc* questionnaire for independent variables and previously validated standardised instruments for dependent variables. The tools used were as follows:

- Sociodemographic Questionnaire for Caregivers: An *ad hoc* instrument collecting data on age, gender, marital status, cohabitation, education level, nationality, employment status, and relationship to the care recipient.
- Connor-Davidson Resilience Scale (CD-RISC 10) (27): This 10-item reduced version assesses resilience in non-professional caregivers using a 5-point Likert scale (0 = “not true at all” to 4 = “true nearly all the time”). Total scores range from 0 to 40, with higher scores indicating greater resilience. The original version demonstrated excellent psychometric properties Cronbach’s alpha (28) was 0.89, with the reduced version showing 0.85 (29) and the Spanish adaptation 0.86. In this study, the Cronbach’s alpha was 0.75, and test-retest reliability yielded r = 0.627.
- Zarit Burden Interview (30, 31): Adapted by Martín et al. (31), this 22-item scale evaluates subjective caregiver burden using a 5-point Likert scale (1 = “never” to 5 = “nearly always”). Scores range from 22 to 110, with cut-off points as follows: 22–46 = no burden, 47–55 = burden, 56–110 = severe burden. Internal consistency in this study was Cronbach’s alpha 0.87, with test-retest reliability of r = 0.76.
- Coping with Stress Questionnaire (CSQ) (32): This 42-item tool assesses behavioural and cognitive coping strategies through seven subscales: problem-solving focus (FSP), negative self-focus (AFN), positive re-evaluation (REP), open emotional expression (EEA), avoidance (EVT), social support seeking (BAS), and religion (RLG). Items are rated on a Likert scale (0 = “never” to 4 = “almost always”) based on experiences over the past year. The CSQ does not have an overall score; instead, the subscales are evaluated independently. Cronbach’s alpha ranged from 0.64 to 0.92 in the original version (mean = 0.79) and from 0.60 to 0.90 in this study (mean = 0.73). Test-retest reliability for each subscale was: FSP (r = 0.79), AFN (r = 0.75), REP (r = 0.62), EEA (r = 0.65), EVT (r = 0.83), and RLG (r = 0.85).
- Medical Outcomes Study Social Support Survey (MOS) (33, 34): This 20-item scale evaluates perceived support from both familial and non-familial networks. Validated in Spanish primary care settings by Revilla et al. (34), it includes one item on social network size and 19 items assessing emotional support, instrumental support, social/leisure companionship, and affective support. Items are rated on a 5-point Likert scale (1 = “never” to 5 = “always”), with a global support index ranging from 19 to 95. The original version reported a Cronbach’s alpha of 0.97. In this study, Cronbach’s alpha was 0.96, and test-retest reliability was r = 0.87.

### 2.4 Data collection procedure

Primary caregivers of people with dementia affiliated with the Alzheimer’s Association were contacted by telephone and invited to participate in the study. In the initial phase, inclusion criteria were verified. Participants were then provided with detailed information about the study objectives, the voluntary nature of participation, and the confidentiality conditions.

Caregivers who agreed to participate signed an informed consent form and subsequently completed a set of self-administered paper-based questionnaires at three time points (T1–T2–T3):

- Sociodemographic questionnaire
- Connor-Davidson Resilience Scale (CD-RISC10)
- Zarit Burden Interview
- Coping with Stress Questionnaire (CSQ)
- Medical Outcomes Study Social Support Survey (MOS)

### 2.5 Intervention procedure

In line with the methodological approach of previous studies (7–9, 25, 26, 35), the intervention consisted of one 90-minute weekly session over six consecutive weeks. Sessions followed an active, participatory methodology grounded in experiential learning and group dynamics, facilitated by a clinical psychologist with specialized training in psychoeducational interventions for family caregivers. Prior to implementation, the facilitator received a two-hour briefing on session structure, objectives, and materials to ensure methodological fidelity and uniform delivery, as proposed Cucco García et al. (36). The intervention was delivered in community settings of local Alzheimer’s Associations, providing a familiar and supportive environment for participants. Each group included 6–10 caregivers. A printed manual and PowerPoint slides guided session content, complemented by handouts summarizing key concepts and reflective exercises for home practice. Attendance was recorded at each session, and participants were required to attend at least three of the six sessions to be considered program completers.

The programme (see **Table 1**) was multicomponent in nature, integrating psychoeducation, emotional support, and skill-building components.

**Table 1:** Structure and objective of each session.

Session structure		
1. Welcome 2. Presentation of the topic 3. Introductory activity related to the topic 4. Explanation of the session’s objective 5. Relaxation technique		
Session	Main objective	Key activities/techniques
1. Initial contact	Introduce the health programme, establish group norms, and explain session dynamics.	Presentation, group ice-breaker, relaxation exercise.
2. Dementia and resilience	Provide information about dementia and introduce the concept of resilience.	Psychoeducation, guided reflection, group discussion.
3. Feelings and emotions	Facilitate emotional awareness and recognition of the caregiver’s emotional responses.	Identification of emotions, sharing experiences, breathing techniques.
4. Leadership and boundaries	Promote self-leadership in caregiving and boundary-setting with the environment.	Role-playing, assertive communication, mindfulness-based exercise.
5. Weaknesses and resources	Encourage self-acceptance and recognition of personal and external support resources.	Strengths inventory, social network mapping, relaxation technique.
6. Healthy habits	Raise awareness of the importance of self-care and adopting healthy routines.	Group brainstorming, habit tracking, relaxation technique.

Fidelity and adherence were monitored through a standardized checklist completed by the facilitator at the end of each session, verifying completion of planned activities and adherence to the session protocol. Periodic supervision by the research team ensured consistency across groups. Participant attendance and engagement (participation in discussions and exercises) were used as indicators of adherence.

Minor procedural adjustments were made during delivery to accommodate participant schedules and group pace (e.g., extending discussion time in sessions 3 and 5). No modifications were made to the core structure, contents, or objectives of the programme, ensuring consistency and fidelity across all groups.

### 2.6 Data analysis

The dataset was initially processed using Microsoft Excel^®^ and subsequently analysed using the statistical software SPSS version 28.0.

Descriptive statistics were used to analyse sociodemographic variables such as age, sex, marital status, household composition, education level, nationality, relationship to the care recipient, employment status, and the number of hours dedicated to caregiving. Inferential statistics were applied to examine resilience, caregiver burden, stress coping strategies, and perceived social support.

Multiple comparisons across the three time points (T1–T2–T3) were conducted within the intervention group to assess the effectiveness of the intervention using the Wilcoxon signed-rank test. Effect sizes were calculated using Rosenthal’s r, and relationships between variables were analysed using Spearman’s Rho correlation coefficient.

Results were interpreted using a 95% confidence interval, and statistical significance was set at p < 0.05.

Given the exploratory nature of the study and the small sample size, resilience was defined as the primary outcome variable. Non-parametric Wilcoxon signed-rank tests were applied, and multiple comparisons were interpreted cautiously to minimize the risk of Type I error. Effect sizes (Rosenthal’s r) and 95% confidence intervals were also calculated to enhance result interpretability.

### 2.7 Ethical and legal considerations

This study was approved by the Ethics Committee of the University of León (ETICA-ULE-035-2020). It adhered to the ethical principles for medical research involving human subjects outlined in the Declaration of Helsinki (37). Since 2016, the Declaration of Taipei (38) complements these principles, addressing ethical considerations related to health databases and biobanks, including the use of information and biological material generated through healthcare provision.

Caregivers were informed both verbally and in writing about the study objectives, the nature of the intervention programme, the data collection procedures, and their rights as participants. They were also provided with a guide outlining the schedule, questionnaire content, and conditions of voluntariness, anonymity, and confidentiality. The informed consent form was signed prior to the commencement of any study-related activity.

Participation in the intervention could be voluntarily withdrawn at any time. Additionally, participants were excluded if they missed more than half of the scheduled sessions.

## 3 Results

**Figure 1:**
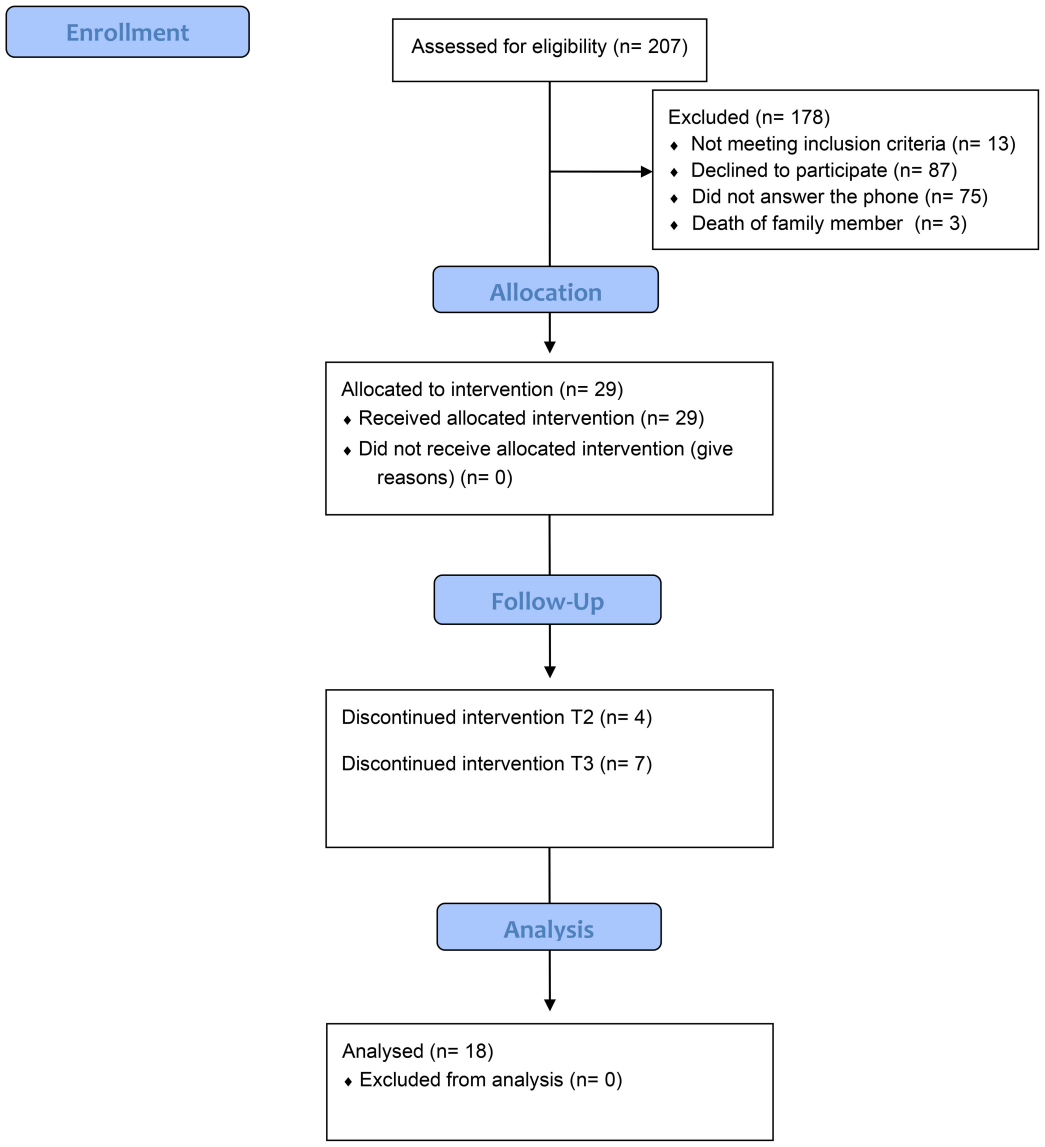
Recruitment and selection participants.

A total of 207 phone calls were made, resulting in the recruitment of 29 female participants. Of these, 25 caregivers completed the post-intervention assessment, representing a 13.79% attrition rate. At the 3-month follow-up, an additional 7 participants were lost, corresponding to a further 24.14% drop-out (see **Figure 1**).

Attrition analyses comparing completers (n = 18) and non-completers (n = 11) revealed no significant baseline differences in age, caregiver burden, or resilience (p > 0.05), suggesting that participant attrition did not introduce systematic bias. Missing data were handled using listwise deletion, as attrition occurred primarily at the final follow-up (T3).

The initial sample consisted of 29 women, with a mean age of 67.97 years (SD = 12.02). The typical caregiver profile was that of a married woman (69.0%), primarily daughters (55.2%) or wives (44.8%) of the care recipient, all of Spanish nationality (100%) and mostly retired (65.5%). Most reported dedicating between 18 and 24 hours per day to caregiving (75.9%). Educational levels varied, with the most common being incomplete primary education (20.7%) and university education (20.7%) (see **Table 2**).

**Table 2:** Descriptive statistics for sociodemographic variables (n=29).

Variable		n (%)
Marital status		
	Single	5 (17.2%)
	Married	20 (69.0%)
	Widowed	2 (6.9%)
	Separated	1 (3.4%)
	Divorced	1 (3.4%)
Living together as a couple		
	Yes	23 (79.3%)
	No	6 (20.7%)
Academic level		
	No education	0 (0%)
	Incomplete primary education	6 (20.7%)
	Primary education or equivalent	5 (17.2%)
	Secondary education	3 (10.3%)
	High school education	5 (17.2%)
	Intermediate vocational training or equivalent	3 (10.3%)
	Advanced vocational training or equivalent	1 (3.4%)
	University education or equivalent	6 (20.7%)
Nationality		
	Spanish	29 (100%)
	Foreign	0 (0%)
Employment status		
	Working	4 (13.8%)
	Unemployed	6 (20.7%)
	Retired	19 (65.5%)
Working hours		
	Full-time	4 (100%)
Family relationship		
	Children	16 (55.2%)
	Spouse	13 (44.8%)
	Nephew	0 (0%)
	Son-in-law/daughter-in-law	0 (0%)
Hours devoted to caregiving		
	0–6 hours	4 (13.8%)
	6–12 hours	2 (6.9%)
	12–18 hours	1 (3.4%)
	18–24 hours	22 (75.9%)

Regarding the results obtained from the administration of the CD-RISC 10, Zarit, CSQ, and MOS questionnaires (**Table 3**), and considering the sample analysed (*n* = 29), the following findings were observed the mean score for resilience was 23.90 (SD = 6.085), suggesting a moderate level of resilience among participants. In contrast, the mean caregiver burden score was 65.38 (SD = 13.963), indicating a high perceived burden related to caregiving responsibilities.

**Table 3:** Descriptive statistics for CD-RISC 10, Zarit, CSQ, and MOS.

	Rank	Minimum	Maximum	Mean	SD
CD-RISC 10	26	7	39	23.90	6.085
ZARIT	51	41	92	65.38	13.963
FSP	22	0	22	12.07	5.451
AFN	17	3	20	10.90	4.678
REP	16	3	19	11.79	4.362
EEA	18	2	20	7.21	3.895
EVT	15	2	17	8.38	4.048
BAS	19	5	24	12.90	6.108
RLG	19	0	19	8.76	6.254
MOS	60	35	95	65.83	17.839
Emotional support	26	14	40	27.97	8.166
Material or instrumental help	13	7	20	12.76	4.665
Social relationships	13	7	20	13.55	3.987
Affectionate support	11	4	15	11.55	3.418

CD-RISC 10 = Connor-Davidson Resilience Scale; ZARIT = Zarit Burden Interview; FSP = focused on solving the problem; AFN = negative self-focus; REP = positive re-evaluation; EEA = open emotional expression; EVT = avoidance; BAS = seeking social support; RLG = religion; MOS = Medical Outcomes Study; SD = standard deviation.

In terms of coping strategies, problem-solving approaches were most frequently used. Specifically, seeking social support had the highest mean score (M = 12.90; SD = 6.108), followed by problem-focused coping (M = 12.07; SD = 5.451).

The mean perceived social support score (MOS) was 65.83 (SD = 17.839), indicating a moderate-to-high level of perceived support.

In line with the analytical plan, resilience was identified as the primary outcome variable. Given the small sample size and non-normal distribution of the data, non-parametric Wilcoxon signed-rank tests were considered appropriate. Multiple comparisons were interpreted with caution to minimize the risk of Type I error. Effect sizes (Rosenthal’s r) and 95% confidence intervals were reported alongside p-values to enhance interpretability.

To analyse changes over time, the Wilcoxon signed-rank test was used for pairwise comparisons across the three time points: T1 (n = 29), T2 (n = 25), and T3 (n = 18). Effect sizes were calculated using Rosenthal’s *r*. The results are presented in **Table 4**, highlighting statistically significant differences in the following comparisons:

**Table 4:** Evolution of scores during the follow-up period.

Instrument/subscale	T1 mean (SD)	T2 mean (SD)	T3 mean (SD)	T1–T2 p	T2–T3 p	T1-T3 p	95% CI	r
Resilience								
CD-RISC-10	23.24 (7.31)	26.36 (6.59)	26.94 (6.55)	*p* = .036	*p* = .101	*p* = .040	[0.02, 0.68]	0.31
Caregiver burden								
ZARIT	65.38 (13.96)	66.48 (13.63)	60.44 (11.59)	*p* = .618	*p* = .033	*p* = .295	[–0.12, 0.54]	0.28
Coping strategies								
FSP	12.07 (5.45)	13.68 (5.27)	14.56 (4.53)	*p* = .579	*p* = .032	*p* = .021	[0.01, 0.60]	0.35
AFN	10.90 (4.68)	9.44 (3.45)	9.33 (3.93)	*p* = .082	*p* = .954	*p* = .119	[–0.15, 0.47]	0.22
REP	11.79 (4.36)	12.12 (4.32)	12.39 (4.15)	*p* = .818	*p* = .703	*p* = .677	—	0.05
EEA	7.21 (3.89)	6.08 (3.43)	5.44 (2.73)	*p* = .011	*p* = .958	*p* = .041	[0.03, 0.62]	0.33
EVT	8.38 (4.05)	9.44 (4.16)	8.24 (4.67)	*p* = .143	*p* = .568	*p* = .909	—	0.09
BAS	12.90 (6.11)	14.04 (6.00)	12.28 (6.18)	*p* = .473	*p* = .120	*p* = .153	—	0.12
RLG	8.76 (6.25)	7.35 (6.27)	3.31 (6.09)	*p* = .026	*p* = .833	*p* = .072	[–0.04, 0.56]	0.26
Social support (MOS)								
Total score	65.83 (17.84)	67.96 (19.10)	66.61 (17.38)	*p* = .219	*p* = .288	*p* = .438	—	0.08
Emotional support	27.97 (8.17)	29.16 (8.32)	28.78 (7.35)	*p* = .359	*p* = .293	*p* = .244	—	0.11
Material help	12.76 (4.67)	13.24 (4.74)	12.72 (4.85)	*p* = .959	*p* = .552	*p* = .975	—	0.04
Social relationships	13.55 (3.99)	13.76 (4.93)	13.61 (3.96)	*p* = .261	*p* = .260	*p* = .734	—	0.06
Affectionate support	11.55 (3.42)	11.80 (3.20)	11.50 (3.35)	*p* = .752	*p* = .452	*p* = .605	—	0.05

T1 = baseline; T2 = post-intervention; T3 = three-month follow-up. p-values are unadjusted Wilcoxon signed-rank test results. CD-RISC-10 = Connor-Davidson Resilience Scale; ZARIT = Zarit Burden Interview; FSP = focus on solving problems; AFN = negative self-focus; REP = positive re-evaluation; EEA = emotional expression; EVT = avoidance; BAS = seeking social support; RLG = religion; MOS = Medical Outcomes Study Social Support Survey; SD = standard deviation; r = effect size. *p <.05 *.

- Resilience (CD-RISC 10): T1–T2 (*p* = 0.036), *r* = 0.29; T1–T3 (*p* = 0.040), *r* = 0.30, both with medium effect sizes.
- Caregiver burden (Zarit): T2–T3 (*p* = 0.033), *r* = 0.33, medium effect size.
- Coping strategies (CSQ): problem-focused coping: T2–T3 (p = 0.032), r = 0.33; T1–T3 (p = 0.021), r = 0.37; open emotional expression: T1–T2 (p = 0.011), r = 0.35; T1–T3 (p = 0.041), r = 0.30; religious coping: T1–T2 (p = 0.026), r = 0.30. All showing medium effect sizes.

A *post hoc* power analysis conducted using G*Power 3.1 indicated that, given the final sample size (n = 18) and the observed medium effect sizes (r ≈ 0.30), the study achieved an approximate statistical power of 0.70, suggesting moderate sensitivity for detecting within-group changes.

As for correlations based on programme follow-up, Spearman’s Rho was calculated for all three time points:

- At T1, a moderate positive correlation was found between resilience (CD-RISC 10) and positive reappraisal (r = 0.497; p = 0.006).
- At T2, moderate negative correlations were observed between resilience and self-focused negative coping (r = -0.437; p = 0.029) and religious coping (r = -0.461; p = 0.020). In contrast, moderate positive correlations emerged between resilience and problem-focused coping (r = 0.431; p = 0.031), as well as with positive reappraisal (r = 0.596; p = 0.002).
- At T3, resilience was positively correlated with problem-focused coping (r = 0.618; p = 0.006), positive reappraisal (r = 0.623; p = 0.006), total perceived social support (MOS) (r = 0.515; p = 0.029), and emotional support subdimension (r = 0.484; p = 0.042).

## 4 Discussion

The present study aimed to evaluate the effectiveness of a group intervention programme designed to foster resilience among primary caregivers of individuals with dementia, as well as to assess the sustainability of its effects three months post-intervention.

The findings suggested a progressive increase in resilience levels among caregivers across the follow-up period, indicating a potential beneficial effect of the programme. These results should be interpreted as preliminary evidence rather than proof of effectiveness. These results are consistent with those of previous (7, 8, 25, 39), which support the usefulness of psychoeducational interventions focused on coping strategies and emotional regulation. However, contrary evidence also exists, such as the study by Kor et al. (40), which found no statistically significant changes in resilience following a comparable intervention.

Regarding the burden associated with caregiving, the results showed a decrease in perceived burden scores. Nonetheless, as Poudevida et al. (8) point out, the progressive course of the disease inevitably leads to an increased caregiving burden over time, due to the escalating care demands.

Previous research (14, 16, 25, 41) has demonstrated that educational programmes aimed at enhancing resilience are effective in improving coping mechanisms in contexts of high emotional burden and prolonged stress. This evidence aligns with the present findings: although the caregivers already employed personal coping strategies, they showed a proactive willingness to adopt new resources throughout the programme. This active engagement appeared to be associated with a moderate increase in resilience (r ≈ 0.30, 95% CI [0.10–0.45]), primarily linked to problem-focused strategies, while passive responses tended to decline.

Correlation analyses indicated a shift toward more adaptive coping strategies, suggesting that changes in coping and perceived support may have coincided with the intervention period. Initially, resilience was associated with positive reappraisal, but post-intervention it also showed positive relationships with problem-focused coping and negative associations with maladaptive styles. At the three-month follow-up, these associations were maintained and complemented by correlations with perceived social support, indicating the consolidation of psychological resources that may facilitate caregivers’ adaptation to the demands of dementia care. From an integrative perspective, the components of the programme, psychoeducation, active coping training, emotional regulation, and strengthening of social support, appear to have operated sequentially across different levels of outcomes. Proximal improvements were reflected in the adoption of more adaptive coping strategies, which likely contributed to an intermediate enhancement in resilience, understood as the capacity to maintain emotional and functional balance in the face of caregiving demands. Ultimately, the reduction in perceived burden represents a distal outcome derived from the sustained improvement in coping and resilience. These findings are consistent with previous evidence (42, 43), which highlights the key role of perceived social support and the caregiver’s social environment in the consolidation of resilience, particularly under persistent emotional and physical demands.

This pattern is consistent with a potential mediational model, which should be empirically tested in future longitudinal studies. Similar pathways have been described in prior research, where coping and resilience sequentially contributed to caregiver adaptation and decreased burden (44, 45). Future research could empirically explore this chain of effects through longitudinal mediation analyses, in order to determine the directionality and magnitude of these relationships, as well as the long-term sustainability of the benefits.

From a temporal and theoretical perspective, the components of the programme, based on psychoeducation, active coping training, emotional regulation, and the strengthening of social support, appear to have influenced outcomes through a sequential mechanism. The psychoeducational and skills-based sessions initially targeted proximal processes by promoting adaptive coping strategies and emotional regulation, which are central mechanisms for managing caregiving stress (46). These proximal changes likely facilitated an intermediate enhancement in resilience—understood as the ability to maintain psychological balance and functional adaptation despite chronic stressors (47, 48). Subsequently, improvements in resilience may have contributed to distal outcomes, such as reductions in perceived caregiver burden (49). This proposed pathway—coping as a proximal mediator of resilience, and resilience as a mediator of burden reduction—offers a coherent explanatory model for the observed effects. Although mediation was not formally tested in this study, future research should examine this hypothesized mechanism using longitudinal and mediation analyses to determine the strength and temporal direction of these associations.

On the other hand, as noted by Poudevida et al. (8), health programmes aimed at improving caregiver wellbeing rarely offer continued support post-intervention to facilitate the implementation and consolidation of learning. Consequently, the benefits achieved tend to diminish over time.

Although not the focus of the present analysis, it is important to acknowledge the difficulties many caregivers face in maintaining adherence to such programmes. As McManus et al. (26) observed, the progression of the disease significantly increases care demands and hospital admissions, which are among the main reasons for absenteeism during follow-up in this type of intervention.

The interpretation of the results should be undertaken with caution due to the small sample size, which limits statistical power and increases susceptibility to selection bias and type II errors. Consequently, the present study should be considered primarily as a feasibility intervention, providing preliminary indications of potential benefit rather than definitive evidence of effectiveness. This limitation poses a direct threat to internal validity, as it reduces the ability to attribute observed effects solely to the intervention. The absence of random allocation prevents the establishment of firm causal relationships; therefore, the identified differences should be regarded as tentative associations rather than demonstrated effects of the programme. Furthermore, the initial sample exhibited moderate levels of resilience, which may have acted as a confounding variable by influencing the magnitude of the improvements observed.

During the follow-up period, a progressive reduction in participant numbers was observed, exceeding initial estimates. This represents an additional threat to internal validity related to experimental mortality, as participant dropout may not have occurred at random. Consequently, the findings should be interpreted with caution, acknowledging the potential for bias in the estimation of change. Nevertheless, the observed differences, though statistically significant with medium effect sizes (r ≈ 0.30, 95% CI [0.12–0.45]), should be considered preliminary and exploratory. These findings tentatively suggest a possible beneficial association with the intervention. Future studies, preferably employing randomised controlled trials with larger sample sizes, are recommended to strengthen causal robustness and the generalisability of the findings.

Additionally, the use of a single-group pretest–posttest design without a control condition further limits internal validity and restricts causal inference. Observed changes might not solely reflect intervention effects but could also be influenced by temporal adaptation, repeated testing, or social desirability bias. Moreover, the exclusive use of self-reported measures may have introduced response bias. Furthermore, it should be noted that participation in a group setting may have produced beneficial effects, such as emotional relief, validation, or a sense of camaraderie, regardless of the specific components of the programme. Therefore, some of the improvements observed could be attributed to participation in a group intervention per se. These potential threats to internal validity should be considered when interpreting the results, which represent associations rather than definitive causal relationships.

In addition, the sample was composed exclusively of Spanish women recruited through Alzheimer’s associations. This lack of gender, cultural, and contextual diversity limits the external validity of the findings and introduces a potential sampling bias. As a result, the transferability of the results to other caregiving populations, such as male caregivers, individuals from different cultural backgrounds, or those unaffiliated with support organizations, remains uncertain. Future studies should aim to include a more diverse and representative sample of caregivers to enhance the generalizability of findings and better understand the differential impact of such interventions across diverse sociodemographic groups.

## 5 Conclusions

The findings of this study provide evidence suggesting a potential beneficial effect of the proposed intervention in enhancing resilience among caregivers, which may contribute to alleviating some of the negative consequences of caregiving on their well-being. Overall, the results suggest that the resources introduced during the sessions were internalized and applied in the caregivers’ daily routines.

Among the study’s key observations, it was noted that women from the immediate family circle most frequently assumed the role of primary caregivers in this sample. Their proactive attitude toward managing caregiving challenges also appeared evident, as several participants reported engaging in problem-solving strategies to cope with daily demands.

The benefits gained from participation in such interventions tend to diminish over time. In light of this, the current findings may support the potential value of implementing programmes in a more continuous and flexible format, which could facilitate the consolidation of learning and enable caregivers to participate according to the progression of their relative’s dementia.

Although the outcomes of this study were generally positive, there remains a notable lack of empirical evidence regarding the relationship between such interventions and the development of caregivers’ strengths in managing their caregiving responsibilities. This research may represent an initial step that contributes to the design of future programmes supported by public administrations, aimed at enhancing caregivers’ resilience. Such initiatives could, in turn, enable the implementation of more studies and help strengthen the emerging evidence base on the long-term effects of these interventions.

## Data Availability

The raw data supporting the conclusions of this article will be made available by the authors, without undue reservation.
